# Recombinant Lysostaphin Protects Mice from Methicillin-Resistant *Staphylococcus aureus* Pneumonia

**DOI:** 10.1155/2014/602185

**Published:** 2014-07-16

**Authors:** Chen Chen, Huahao Fan, Yong Huang, Fan Peng, Hang Fan, Shoujun Yuan, Yigang Tong

**Affiliations:** ^1^State Key Laboratory of Pathogen and Biosecurity, Beijing Institute of Microbiology and Epidemiology, 20 Dong-Da Street, Fengtai District, Beijing 100071, China; ^2^Department of Pharmacology and Toxicology, Beijing Institute of Radiation Medicine, 27 Taiping Road, Haidian District, Beijing 100850, China; ^3^Anhui Medical University, Hefei 230032, China; ^4^Laboratory of Gene Regulation, Faculty of Medicine, University of Tsukuba, Tsukuba 305-8575, Japan; ^5^Department of Microbiology, Peking University Health Science Center, Beijing 100191, China

## Abstract

The advent of methicillin-resistant *Staphylococcus aureus* (MRSA) and the frequent and excessive abuse of ventilators have made MRSA pneumonia an inordinate threat to human health. Appropriate antibacterial therapies are crucial, including the use of lysostaphin as an alternative to antibiotics. To explore the potential use of lysostaphin as a therapeutic agent for MRSA pneumonia, mice were intranasally infected with MRSA and then treated with recombinant lysostaphin (rLys; 45 mg/kg in the high-dose group and 1 mg/kg in the low-dose group) (0.33 mg/mL, 15 mg/mL), vancomycin (120 mg/kg) (40 mg/mL), or phosphate-buffered saline (PBS, negative control) 4 h after infection. Therapeutic efficacy was assessed by mouse survival, lung histopathology, bacterial density in the lungs, bodyweight, lung weight, temperature, white blood cells counts, lymphocytes counts, granulocytes counts, and monocytes counts. The mice treated with rLys showed lower mortality, less lung parenchymal damage, and lower bacterial density at metastatic tissue sites than mice treated with PBS or vancomycin. The overall mortality was 100%, 60%, 40%, and 60% for the control, vancomycin, high-dose rLys, and low-dose rLys groups, respectively. These findings indicate that, as a therapeutic agent for MRSA pneumonia, lysostaphin exerts profound protective effects in mice against the morbidity and mortality associated with *S. aureus* pneumonia.

## 1. Introduction

*Staphylococcus aureus* is one of the most common human pathogens. Up to 20–30% of carriers are persistently and asymptomatically colonized and 50–60% are intermittently colonized [1].* Staphylococcus aureus* causes many skin and soft-tissue infections and invasive diseases such as sepsis, endocarditis, pneumonia, and osteomyelitis [2]. These infections are complex to treat because this bacterial species can become resistant to antibiotics. At present, methicillin-resistant* S. aureus* (MRSA) is one of the most commonly identified antibiotic-resistant pathogens in many parts of the world. Moreover, MRSA infection rates have increased exponentially worldwide over the past few decades. Most of these infections, including sepsis and pneumonia, are often characterized by fulminant onset, rapid progression, and in a subset of patients, a fatal outcome [3]. Among these invasive infections, necrotizing* S. aureus* pneumonia has emerged as one of the most lethal [4, 5]. The reduced efficacy of vancomycin and linezolid against MRSA has increased the threat of incurable staphylococcal infections [6].

The proportion of MRSA exceeds 10% in the 24 participant countries within the European Antimicrobial Resistance Surveillance System (EARSS) [7]. Moreover, accumulating data indicate that MRSA infections are associated with a worse prognosis than methicillin-susceptible* S. aureus* infections [8–11]. Severe healthcare-associated MRSA infections, including bacteremia, hospital-acquired pneumonia, and ventilator-associated pneumonia, are associated with a particularly high risk of mortality and complications. The optimal therapy for these infections remains a therapeutic challenge.

Lysostaphin is a 27 kDa peptidase produced by* Staphylococcus simulans*, which was isolated in 1964 by Schindler and Schuhardt [12–14]. Lysostaphin specifically cleaves the pentaglycine cross-links unique to the cell wall of* Staphylococci* and lyses cells in all metabolic states (growing, resting). Because* Staphylococci* are highly resistant to lysis with standard agents, such as lysozyme or detergents, lysostaphin has been widely used in research laboratories as a staphylolytic agent. Here, we assessed the therapeutic efficacy of lysostaphin against infection with a clinical MRSA isolate in an animal model and compared its antibacterial efficacy with that of vancomycin.

## 2. Materials and Methods

### 2.1. *Staphylococcus aureus* Isolate

The MRSA isolate strain MRSA-117 used in this study was isolated from the Affiliated Hospital, Academy of Military Medical Sciences (China). The isolate selected was recovered from the sputum of a 72-year-old male patient with pneumonia. MRSA-117 was shown to be resistant to several antibiotics (Table 1).

### 2.2. Production and Purification of Lysostaphin

pQE30-lysostaphin was constructed by subcloning a gene encoding lysostaphin into the pQE30 vector (Invitrogen, China). The protein-coding sequence of lysostaphin was obtained from the National Center for Biotechnology Information (GenBank accession: YP_003505772). The modified lysostaphin gene only contains the functional genes but not signal genes (see Table 2 about the modified lysostaphin gene sequence), and the modified lysostaphin gene was inserted into the pQE30 vector using conventional cloning techniques, with restriction enzymes* Bam*HI and* Hin*dIII. The resulting lysostaphin-expressing plasmid was designated pQE30-lysostaphin.* Escherichia coli* M15 cells transformed with pQE30-lysostaphin were used as the production host for lysostaphin. Actually, the active lysostaphin protein could be expressed by only part of the whole lysostaphin gene, which was synthesized by Invitrogen, and the sequence information was in the Table 2 (5′-*Bam*HI, 3′-*Hin*dIII). The expression of lysostaphin was induced with 1 mmol/L Isopropyl*β*-D-1-Thiogalactopyranoside (IPTG) at an optical density at 600 nm (OD_600_) of 0.6 and the induced bacterial cells were then incubated for an additional 8 h at 37°C with shaking at 200 rpm. The bacterial cells were recovered by centrifugation (6000 ×g for 20 min) and the resulting cell pellet was resuspended in lysis buffer (50 mmol/L NaH_2_PO4 containing 300 mmol/L NaCl, pH 8.0) and disrupted with a conventional ultrasonic treatment for 30 min (2 s pulses with 2 s rest intervals between pulses). Following centrifugation (5000 ×g for 30 min), the supernatant was recovered and subjected to two-step chromatography that included ion-exchange chromatography (SP Sepharose Fast Flow column; GE Healthcare, Sweden) and hydrophobic-interaction chromatography (Toyopearl PPG-600M column; Tosoh Bioscience, Japan).

The endotoxin unit of recombinant lysostaphin (rLys) was determined to be less than 1.0 international endotoxin units (EU)/mL by the clinical laboratory of the Affiliated Hospital, Academy of Military Medical Sciences (Table 3) using both micro-Kjeldahl method and Micro-Ultraviolet Spectrophotometer. And the purity of the rLys was shown to be 90%.

### 2.3. *In Vitro* Antibacterial Activity Test

The* in vitro* antibacterial activity of the rLys against MRSA was investigated with the double AGAR plate method. Various concentrations of lysostaphin were dropped onto the culture of double AGAR plate. The bacteria were allowed to grow for 8 h after treatment with lysostaphin, and the plates were then examined to determine whether the bacterial growth was inhibited by lysostaphin.

### 2.4. MRSA Infection and Lysostaphin Treatment in a Mouse Model

To prepare an animal inoculum, a frozen stock of MRSA-117 was subcultured on trypticase soy agar and cultured overnight at 37°C. Trypticase soy broth (TSB; 5 mL) was inoculated with a single colony and was cultured overnight at 37°C with shaking at 200 rpm. After 100-fold dilution, the overnight culture was grown in fresh TSB and incubated for about 3 h 37°C at 200 rpm (OD_600_ = 1.0). The bacteria were centrifuged at 10 000 ×g for 10 min, washed, and resuspended in sterile phosphate-buffered saline (PBS). This process was repeated twice, and the bacterial suspension was adjusted to a final density of 1 × 10^10^ colony-forming units (CFU)/mL (6 × 10^8^ CFU per 60 *μ*L).

Six-week-old specific-pathogen-free (SPF) female BALB/c mice were obtained from the Experimental Animal Center of the Academy of Military Medical Sciences and maintained in a biosafety level 2 facility. The animal experiments were conducted in accordance with the regulations for laboratory animals of the Ministry of Science and Technology. The mice were immunosuppressed with 200 mg/kg cyclophosphamide (CTX, Baxter Oncology GmbH, Germany) injected intraperitoneally for two consecutive days before infection. The mice were then anesthetized with sodium pentobarbital, hung in an upright position, and inoculated intranasally with 60 *μ*L of MRSA-117 suspension (6 × 10^8^ CFU). After inoculation, the animals were held upright for 30 s. Four hours after infection with MRSA-117, the mice were anesthetized again with sodium pentobarbital and intranasally administered 60 *μ*L of rLys (two dosage groups: 45 mg/kg and 1 mg/kg), vancomycin (VAN; 120 mg/kg), and PBS. The intrarectal temperature of the infected mice was monitored with an electronic thermometer (MC-246, Omron, Japan).

To determine the pathological correlations of staphylococcal pneumonia, the weigh, illness condition and mortality of the infected mice were recorded daily for 30 days after infection. And the infected mice were euthanized with cervical dislocation under anesthesia, their lungs were weighed and homogenized to calculate the bacterial burden by the double AGAR plate method.

### 2.5. Mouse White Blood Cell (WBC) Counts

Peripheral blood samples obtained from the tails of the mice were collected in heparin-coated quantitative blood collection tubes. The samples were analyzed with an M16 Medonic automated cell-counting instrument (Medonic, Sweden). The results are presented as mean values ± standard deviations.

### 2.6. Histology

After the mice were killed, their lungs were excised and weighed. They were then washed in PBS and inflated with 4% buffered formalin, sequentially infiltrated with increasing concentrations of ethanol and xylene, and embedded in paraffin. The tissues were then sectioned, stained with hematoxylin and eosin, and visualized with microscopy.

### 2.7. Statistical Analysis

All data are given as mean values and standard deviations. Survival curves were created using the Kaplan-Meier method and compared with a log-rank (Mantel-Cox) test. Weight loss, temperature loss, and blood cell data were compared with student's *t*-test in the Origin version 8.0 software. A value of *P* < 0.05 was considered to indicate a significant difference.

### 2.8. Ethical Approval

All animal work was approved by the Animal Ethics Committee of the Beijing Institute of Microbiology and Epidemiology (permit number: SCXK-(JUN) 2007-004).

## 3. Results

### 3.1. Production and Purification of Lysostaphin

**Figure 1:**
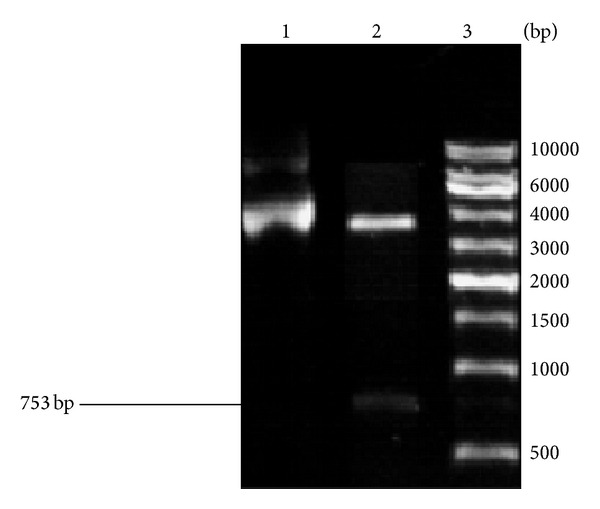
Restriction enzyme digestion of the constructed plasmid pQE30-lysostaphin. Line 1: plasmid pQE30 (3461 bp); line 2: plasmid pQE30-lysostaphin digested with* Bam*HI and* Hin*dIII, producing fragments of 3461 bp (vector plasmid pQE30) and 753 bp (*Lys* gene), respectively; line 3: DNA molecular weight marker.

**Figure 2:**
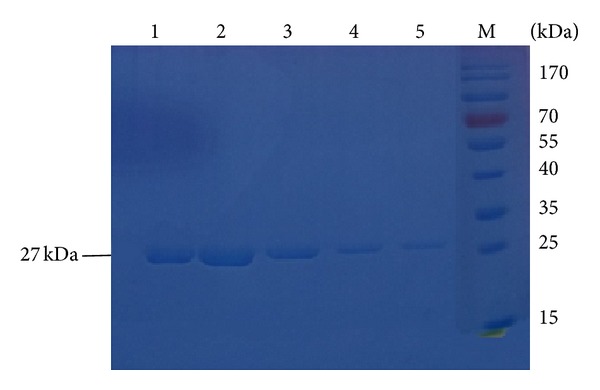
SDS-PAGE analysis of the purified recombinant protein. M: protein molecular weight marker; lines 1–5: five different purified His-rLys protein samples.

The lysostaphin gene was inserted into the pQE30 vector using conventional cloning techniques with the restriction enzymes* Bam*HI and* Hin*dIII (Figure 1), and the rLys was successfully expressed in a soluble form, as demonstrated by sodium dodecyl sulfate polyacrylamide gel electrophoresis (SDS-PAGE; Figure 2). rLys was expressed with a yield of 90.7 mg/L of culture.

### 3.2. *In Vitro* Antibacterial Properties of rLys

**Figure 3:**
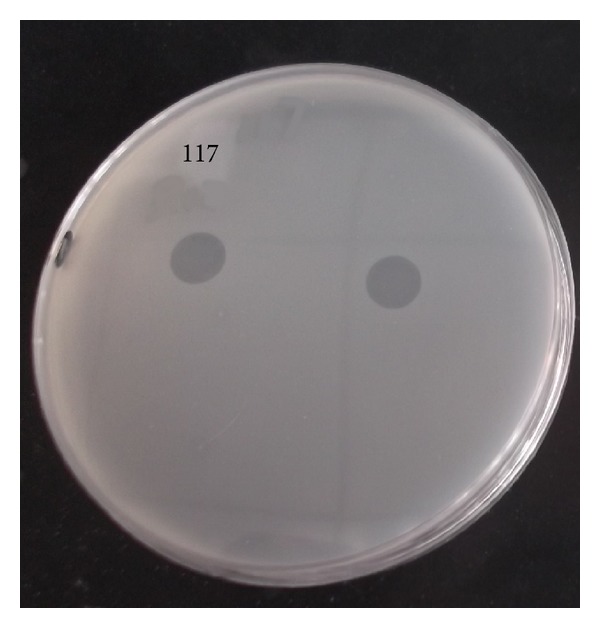
*In vitro* activity of rLys. rLys was dropped onto a plate containing MRSA-117, which was then incubated at 37°C for 8 h. The left one is 1 *μ*L 1 mg/kg rLys and the right one is 1 *μ*L 45 mg/kg rLys; both kinds of rLys are active enough to lyse the bacteria* in vitro*.

Recombinant lysostaphin was dropped onto a plate inoculated with MRSA-117 when the bacterial lawn formed; a clear circular “halo” (zone of inhibition) appeared around the area upon which the lysostaphin had been dropped, indicating that rLys has an antimicrobial effect against MRSA strain MRSA-117* in vitro* (Figure 3).

### 3.3. Therapeutic Effects of Lysostaphin in the Mouse Challenge Model

Four groups of animals were inoculated with MRSA-117 in this study. rLys (high and low doses), VAN, or PBS (the negative control) was administered 4 h after inoculation. After their recovery from anesthesia, all the inoculated animals appeared ill within 2 h, with rapid labored breathing. All the mice curled up with one another and were listless, drinking, and eating little.

**Figure 4:**
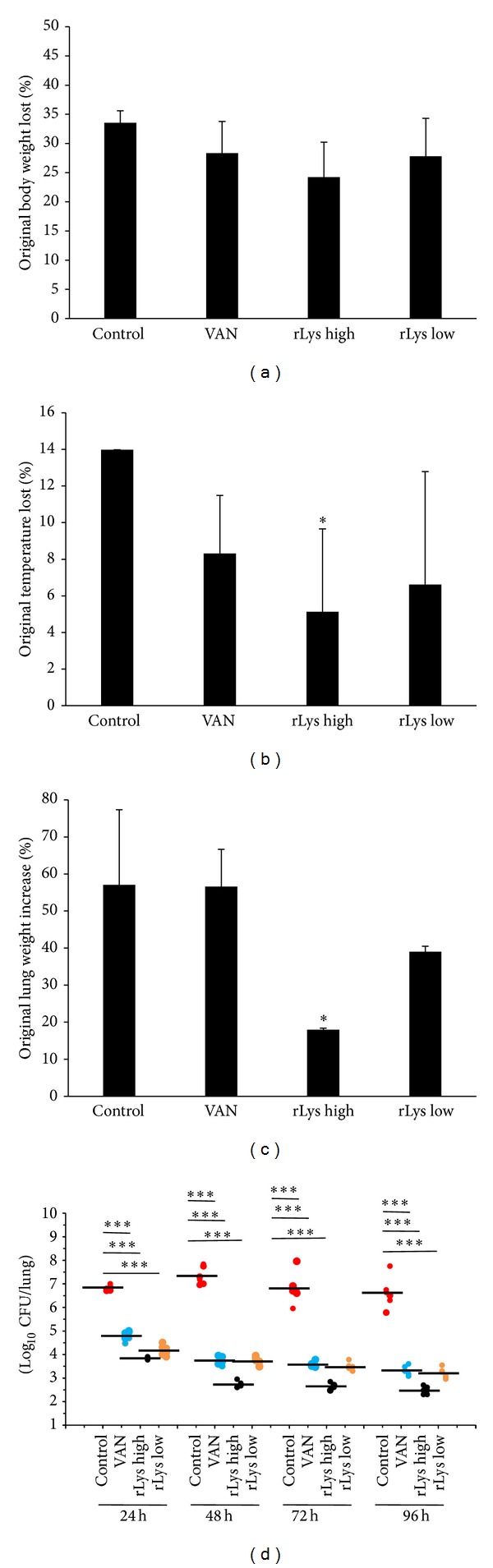
MRSA-infected animals treated with PBS, VAN, high-concentration rLys, or low-concentration rLys. (a) Weight loss in MRSA-infected animals. Mice were weighed 96 h after infection. The mouse bodyweight loss is represented as mean (±SD) of original bodyweight lost (*P < 0.05, Student's *t*-test). (b) Intrarectal temperature was monitored at different time points and the change in temperature between 0 and 96 h (or death) is plotted. (c) Increase in lung weight, represented by the mean (±SD) of original lung weight increase (*P < 0.05, Student's *t*-test). (d) Viable bacterial counts (log_10_ CFU/lung) in the lung homogenates of MRSA-infected mice killed at different time intervals after treatment. Values are expressed as means (±SD) of the original data, *n* = 5. Statistical significance was determined by one-way analysis of variance with the Bonferroni test (*P < 0.05, ***P < 0.001).

The control animals treated with PBS all died within 96 h. The animals treated with rLys or VAN lost less weight than the negative control animals. Mice that received 45 mg/kg rLys lost 24% of their original bodyweight on average, whereas those given 120 mg/kg VAN or 1 mg/kg rLys lost 34% and 28% of their original bodyweights, respectively (Figure 4(a)). The mice in the high-rLys group were less hypothermic and recovered more rapidly than the animals in the negative control group and the other two treatment groups (*P* < 0.05; Figure 4(b)). A histochemical analysis showed that only animals in the high-rLys group were significantly protected from pulmonary edema 96 h after infection (*P* < 0.05), and the protective effects on mice treated with 1 mg/kg rLys or VAN were less obvious than the effects on those treated with 45 mg/kg rLys (Figure 4(c)).

The bacterial burden in the lungs was quantified to assess the influence of rLys on MRSA survival within the mouse lungs. The bacteria content in the lungs of mice treated with 45 mg/kg rLys was significantly lower than those in the PBS group at 24, 48, 72, and 96 h after infection (*P* < 0.001; Figure 4(d)).

**Figure 5:**
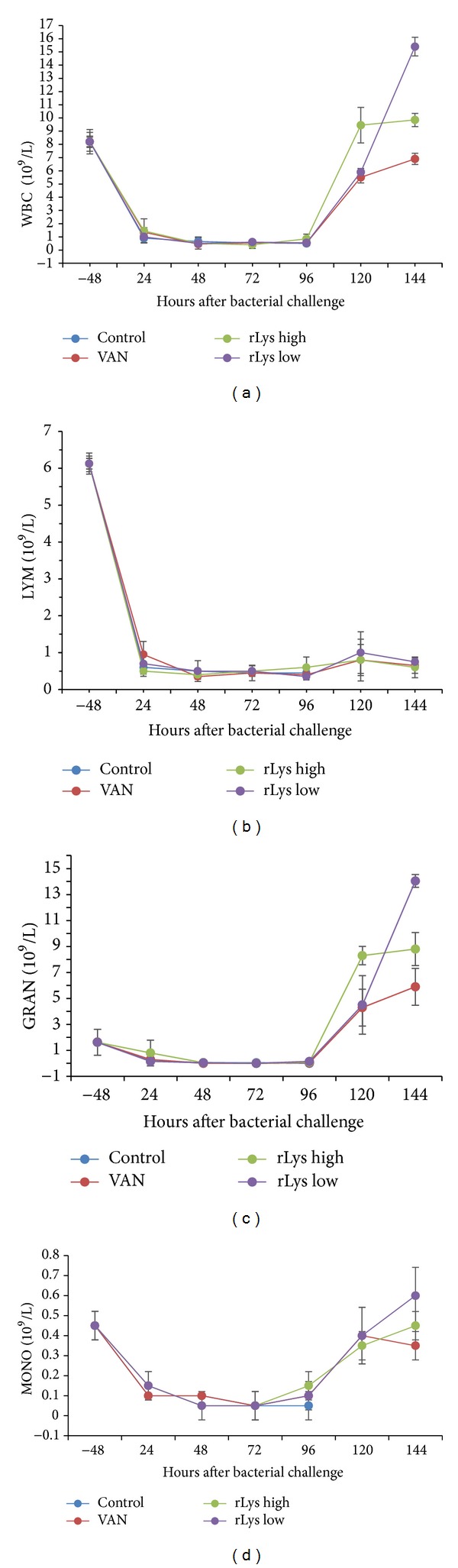
Blood cell analysis in mice at different time intervals after treatment (*n* = 5). (a) White blood cell counts; (b) lymphocyte counts; (c) granulocyte counts; (d) monocyte counts.

To determine whether rLys affects the immune cells, we analyzed the WBC in the peripheral blood of mice receiving the high and low doses of rLys separately. WBC counts were made at early time points, and the mean values and standard deviations of each group are presented in Figure 5(a). The results showed a significant reduction in the WBC counts after the injection of CTX, which indicated that the mice were immunosuppressed by CTX. After the treatments, the groups receiving VAN or low-dose rLys showed a persistent marked increase in WBCs, which were almost restored to the normal range on day 5 after bacterial challenge, whereas the WBC counts in the mice treated with high-dose rLys were even higher, increasing above the normal range. No differences were observed in the WBC nadirs of the treated mice and the control mice. All the treated groups had recovered normal WBC counts by day 5 after infection (Figure 5(b)). Further analysis of the blood cell counts suggested that the reduced WBC counts in the immunosuppressed animals were mainly attributable to a severe reduction in lymphocytes, from an average of 6.1 × 10^9^/L to 4.5 × 10^8^/L (Figure 5(c)). However, the increased WBC counts in the VAN- and rLys-treated mice resulted from substantial increases in granulocytes and monocytes (Figures 5(c) and 5(d)).

**Figure 6:**
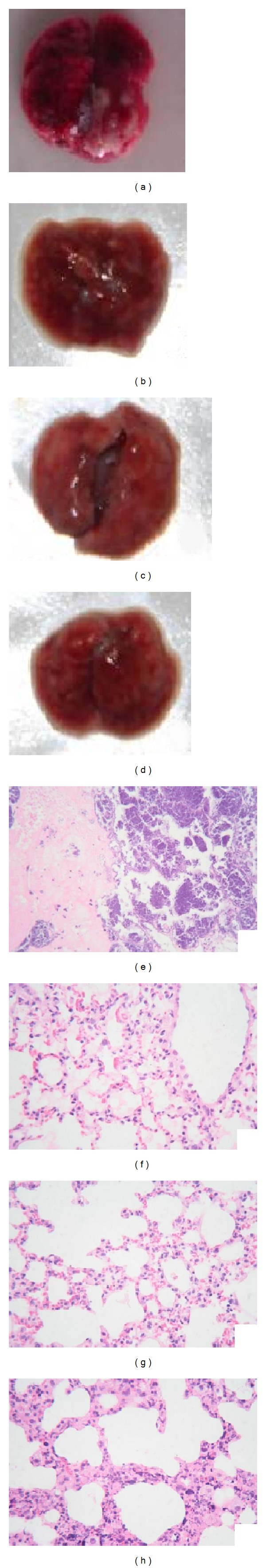
Histopathology of the lungs of untreated (PBS) and treated (VAN or rLys) animals (*n* = 5). (a)–(d) Gross histopathology of the lungs. (e)–(h) Histopathology of the lung tissues. (a) and (e) Control mice treated with PBS. Infection with MRSA showing typical necrotizing pneumonia with multifocal bacterial colonies, complete destruction of the alveolar architecture, hemorrhage, and perivascular growth of* S. aureus*. Presence of acute pneumonia with neutrophils in the distal bronchioles and alveolar spaces is also evident. Suppurative inflammation is present in the interstitium and at perivascular locations. (b) and (f) Lungs from mice infected with MRSA after treatment with VAN. This panel demonstrates the reduced acute inflammation, although it is still apparent as neutrophils and neutrophil debris throughout the lung interstitium, with congestion and intra-alveolar fluid. (c) and (g) Lungs from mice treated with 45 mg/kg rLys. This panel shows the reduced acute inflammation; the lung tissue is similar to normal lung tissue. (d) and (h) Lungs from mice treated with 1 mg/kg rLys. This panel shows the reduced acute inflammation, although inflammation is still evident as neutrophils and neutrophil debris. (e)–(h) Tissues were stained with hematoxylin and eosin (original magnification, ×400).

To evaluate the impact of the rLys treatment on the pathological manifestations of lung injury, we performed a histopathological analysis 96 h after infection of the lungs from mice treated with VAN, high- and low-dose rLys, or PBS (control). Gross inspection indicated that the lung tissues of the infected mice were crimson and had a tight texture. Following treatment with rLys, the lung tissues of the infected mice were light pink, whereas the lungs of the VAN-treated mice showed multifocal inflammatory cell infiltration (Figures 6(a)–6(d)). There was significant accumulation of inflammatory cells (dark blue or purple) in the alveolar spaces after MRSA infection, as shown in Figures 6(e)–6(h). Treatment with high-dose rLys resulted in a marked alleviation of pulmonary inflammation, as indicated by the lower accumulation of cellular infiltrates in the alveolar spaces.

There was evidence of reduced acute inflammation and injury in the lungs of the MRSA-infected mice treated with rLys, especially in the high-dose rLys group, compared with those treated with either PBS or VAN (Figure 6). Hematoxylin-eosin staining of the tissues seldom revealed bacteria in the VAN- or rLys-treated mice (Figures 6(f)–6(h)).

On day 1 after MRSA infections, the mice in the control group (treated with PBS) began to die, and on day 2, the mice in the low-dose rLys treatment group began to die, whereas mice in the other treatment groups (VAN and high-dose rLys) began to die on day 3. There was no death in the blank control group (uninfected) or in the immunocompetent control group (MRSA infected). At the end of the experiment, all the mice in the PBS-treated group had died. Four, six, and three mice survived in the VAN group, high-dose rLys group, and low-dose rLys group, respectively, and the survival rates in each group were 40%, 60%, and 30%, respectively. The mean survival time of each treatment group differed significantly from that of the PBS control group (*P* < 0.01; Table 4). The mice in the treatment groups had longer median survival times and lower death rates than those in the control group. The survival times of the being infected and eventually dead animals in the lysostaphin treatment group were longer than those in the control group or the VAN treatment group (*P* < 0.05). This indicates that lysostaphin delayed death and even protected the animals from death after infection (Table 4).

**Figure 7:**
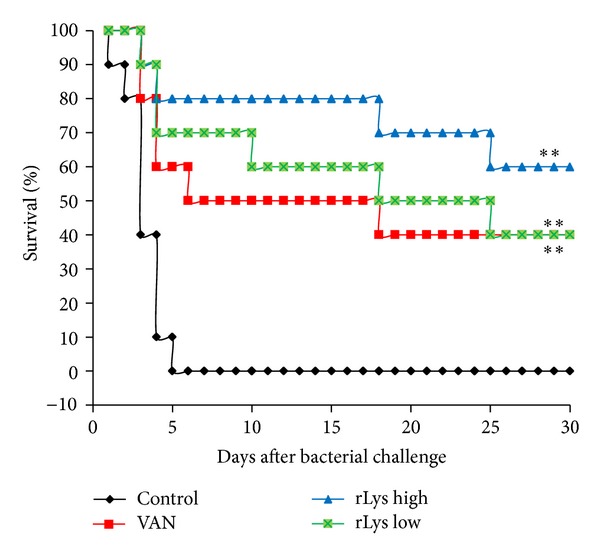
Survival of mice treated with intranasal PBS, rLys (45 mg/kg), rLys (1 mg/kg), or VAN. Each group was monitored for 30 days, and the results are shown as a Kaplan-Meier plot, *n* = 10. Statistical significance was determined with one-way analysis of variance and the Bonferroni test (*P < 0.05, **P < 0.01, ***P < 0.001).

The clinical consequences of intranasal PBS, rLys (45 mg/kg), rLys (1 mg/kg), or VAN treatment were studied in the MRSA-infected mice. The overall survival rate in the control group (PBS; 0% survival rate) was lower than that in the animal groups treated with VAN (40% survival rate), high-dose rLys (60% survival rate), or low-dose rLys (40% survival rate), and these differences were statistically significant (*P* < 0.01). The mice in the control group began to die from day 1 after infection. On day 5, mortality was 100%, 40%, 30%, and 20% for the control, VAN, low-dose rLys, and high-dose rLys groups, respectively. Mice treated with high-dose rLys had increased life spans compared with those of the mice in the control, VAN, and low-dose rLys groups. The survival curve for the high-dose rLys group differed significantly from that of the control group (*P* < 0.01). When the dose was reduced to 1 mg/kg, the survival curve was also markedly different from the control (*P* < 0.01). Compared with the mice treated with PBS, the animals treated with VAN or rLys exhibited significantly better survival, free from severe pneumonia. The mice in the high-dose rLys group were best protected from death compared with the other groups (Figure 7).

## 4. Discussion

The frequency of pneumonia caused by hospital-acquired MRSA (HA-MRSA) and community-acquired MRSA (CA-MRSA) is increasing. CA-MRSA pneumonia is associated with an influenza-like illness, often occurs in young healthy individuals, and results in an acute infection with a stormy course, numerous complications, and high mortality rates. HA-MRSA pneumonia is a frequently fatal illness that occurs in older, debilitated patients, especially those receiving ventilator support. Most cases of MRSA pneumonia are caused by HA-MRSA. With the continuing increase in antibiotic resistance and the decline in the discovery of new antibiotics, we are now entering the “postantibiotic era,” with limited treatment options available for many bacterial infections, including MRSA [15].

The intranasal inoculation of mice with MRSA isolates caused illness with reproducible clinical and pathological features. However histopathological analysis of lung tissues revealed necrotizing pneumonia resembling that documented in postmortem tissues from patients [16] and was characterized by the obliteration of the alveolar architecture, the perivascular accumulation of* S. aureus*, and hemorrhage. The presence of pulmonary hemorrhage is noteworthy because hemorrhage has recently been identified as an important risk factor in predicting mortality in patients with MRSA necrotizing pneumonia [17]. A high level of suspicion, aggressive diagnostic measures, and the rapid application of an effective therapy are essential if we are to improve the mortality rates for these diseases.

Recent studies have shown that MRSA is resistant to almost all the *β*-lactam antimicrobial agents (ceftaroline is an exception) in the present market and is also resistant to 80% of common antimicrobial agents such as gentamicin and the macrolides. Therefore, vancomycin and linezolid are currently recommended for the treatment of clinical MRSA pneumonia [18], but these treatments have been disappointing. With the widespread use of vancomycin, the drift in its minimal inhibitory concentration (MIC) for MRSA has been high, and large numbers of MRSA strains that are resistant to vancomycin have appeared [19, 20]. The significant increase in vancomycin MIC values not only prolongs hospitalization times, but also leads to significant increases in mortality. The sensitivity of MRSA to linezolid has declined, and long-term medication with linezolid (>14 days) can cause adverse events, such as thrombocytopenia [21].

Therefore, rLys may be a better option for the treatment of MRSA pneumonia. Our animal experiments show that lysostaphin can reduce the bodyweight loss and decrease in body temperature associated with MRSA pneumonia in the mouse, indicating that lysostaphin promotes the recovery of the animal's bodyweight and body temperature. Lysostaphin also reduced the number of bacteria in the lungs through its direct bactericidal activity, thereby reducing lung inflammation and reducing the weight increase in the infected lung that occurs during inflammation. All these observations were confirmed by hematoxylin-eosin staining of the mouse lung tissues.

Mouse blood leukocyte counts reflect one aspect of their immune function. The numbers of mouse leukocytes (WBC, lymphocytes, granulocytes, and monocytes) decreased significantly after the injection of the immunosuppressant CTX and continued to decline until death if not treated appropriately. However, these leukocyte numbers began to increase gradually in the mice treated with VAN or rLys and recovered faster in the lysostaphin-treated mice than in the VAN-treated mice. The survival curves showed that the mice in the control group began to die on day 1 after infection, and most deaths occurred 72–96 h after infection. rLys treatment delayed the death of the MRSA-infected mice and increased the overall survival rate of the infected animals. Accordingly, the median survival time and the average survival time of the animals were also improved by treatment with lysostaphin.

By increasing the body weights and temperatures of the mice, lysostaphin treatment enhanced their metabolism, promoting the formation of immune cells and improving the body's defenses, so that the mice could recover rapidly, with greater alleviation of the MRSA pneumonia symptoms, a lower incidence of death, and longer survival times.

Although a few studies have shown lysostaphin to be an effective agent for the treatment of experimental MRSA keratitis and endophthalmitis [22], there have been concerns regarding enzyme degradation and the immunogenicity of lysostaphin in terms of its safety and efficacy. Lysostaphin treatment for MRSA pneumonia has not been studied until now. Here, we explored the treatment of MRSA pneumonia with lysostaphin in mice, and our findings may clarify how lysostaphin protects mice against MRSA infection. Further studies of its efficacy in treating both HA-MRSA pneumonia and CA-MRSA pneumonia are essential.

Several studies [23] have demonstrated that lysostaphin is a novel antistaphylococcal agent for the treatment of* S. aureus* infections. However, there are certain limitations to its use. For instance, a mechanism of resistance to lysostaphin was identified that involves mutations affecting* femA*, which encodes the protein responsible for the addition of the second and third glycines to pentaglycine cross-bridges. Mutations affecting femA renders this protein nonfunctional, resulting in monoglycine cross-bridges rather than pentaglycine bridges [24–29], and this causes* S. aureus* cells to be either partially or completely resistant to lysostaphin.

Lysostaphin is unique among antistaphylococcal agents in that it kills bacteria, whether active or resting, capsulated or noncapsulated, and it is thus capable of killing a large number of organisms in the genus* Staphylococcus* because of the existence of glycine-glycine bonds [30]. More significantly, because the effectiveness of this enzyme against MRSA has been demonstrated* in vivo*, lysostaphin might prove to be useful in the treatment of MRSA infections alone or in combination with antibiotics. Ultimately, it could have potential use in the treatment and prevention of many resistant staphylococcal infections.

In summary, rLys displayed better antibacterial activity against MRSA than VAN* in vitro* and* in vivo*. Its unique specificity, low toxicity, and increasing stability mean that rLys might be a potential agent for the treatment of various* S. aureus* infections in humans. However, more understanding of the structural and functional properties of lysostaphin is required to standardize drug formulations containing lysostaphin either alone or in combination with other antibiotics for use against MRSA and other antibiotic-resistant* S. aureus*.

## Figures and Tables

**Table 1:** Bacterial antibiotic susceptibility testing of MRSA strain MRSA-117.

Antibiotic	MIC (*μ*g/mL)	Sensitivity
Amoxicillin/CA		R
Gentamicin	>=16	R
Imipenem		R
Oxacillin		R
G Penicillin-G	>=0.5	R
Rifampin	>=32	R
Tetracycline	>=16	R
SMZCO	40	S∗
Vancomycin	<=0.5	S∗
Levofloxacin	>=8	R
Azithromycin		R
Ampicillin/sulbactam		R
Clarithromycin		R
Quinupristin/dalfopristin	0.5	S∗
Nitrofurantoin	32	S∗
Linezolid	2	S∗
Moxifloxacin	>=8	R
Tigecycline	<=0.12	S∗

S: sensitive; R: resistant; ∗very sensitive.

**Table 2:** The modified lysostaphin gene sequence.

1 ggatccgctg caacacatga acattcagca caatggttga ataattacaa aaaaggatat
61 ggttacggtc cttatccatt aggtataaat ggcggtatgc actacggagt tgattttttt
121 atgaatattg gaacaccagt aaaagctatt tcaagcggaa aaatagttga agctggttgg
181 agtaattacg gaggaggtaa tcaaataggt cttattgaaa atgatggagt gcatagacaa
241 tggtatatgc atctaagtaa atataatgtt aaagtaggag attatgtcaa agctggtcaa
301 ataatcggtt ggtctggaag cactggttat tctacagcac cacatttaca cttccaaaga
361 atggttaatt cattttcaaa ttcaactgcc caagatccaa tgcctttctt aaagagcgca
421 ggatatggaa aagcaggtgg tacagtaact ccaacgccga atacaggttg gaaaacaaac
481 aaatatggca cactatataa atcagagtca gctagcttca cacctaatac agatataata
541 acaagaacga ctggtccatt tagaagcatg ccgcagtcag gagtcttaaa agcaggtcaa
601 acaattcatt atgatgaagt gatgaaacaa gacggtcatg tttgggtagg ttatacaggt
661 aacagtggcc aacgtattta cttgcctgta agaacatgga ataaatctac taatacttta
721 ggtgttcttt ggggaactat aaagtgaaag ctt

The full lysostaphin gene sequence is 1359 bp, and the modified lysostaphin gene sequence in this work is 753 bp.

**Table 3:** Endotoxin detection in the samples to be used for treatment.

Samples	Result (EU/mL)
O	0.68
H	0.76
L	0.72

O: PBS used for the negative control group; H: rLys (45 mg/kg) used for the high-dose group; L: rLys (1 mg/kg) used for the low-dose group. Negative results: <1 EU/mL; suspicious results: 1-2 EU/mL; positive results: ≥2 EU/mL.

**Table 4:** Survival times of mice.

Group	Time of death (days)		Number of survival animals being infected	Survival (%)	Median survival time of being infected and eventually dead animals (days)	The mean survival time of being infected and eventually dead animals (days)	The mean survival time of being infected and eventually dead animals (days)
	Begin	The finial					
Control	1	5	0	0	3	3.2 ± 1.1	3.2 ± 1.1
VAN	3	18	4	40	4	15.8 ± 13.0∗∗	6.3 ± 5.8
rLys (45 mg/kg)	3	25	6	60	11	23.0 ± 11.0∗∗∗	12.5 ± 10.8∗
rLys (1 mg/kg)	3	25	4	40	7	18.4 ± 12.0∗∗∗	10.7 ± 9.0∗

*P < 0.05, **P < 0.01, and ***P < 0.001 compared with the control group.
